# Noninvasive Outflow Graft Flow Waveform Assessment Using Echocardiography and the HeartMate 3 Snoopy: A Mock Loop Study

**DOI:** 10.1097/MAT.0000000000002610

**Published:** 2025-12-22

**Authors:** Ricardo C. Deveza, Mina Saweris, Desiree Robson, Pankaj Jain, Sumita Barua, Christian Said, Theodore Abart, Gregor Widhalm, Kavitha Muthiah, Thomas Schlöglhofer, Christopher Hayward

**Affiliations:** From the Cardiology Department, * St Vincent’s Hospital, Darlinghurst, Australia; † Faculty of Medicine and Health, School of Clinical Medicine, University of New South Wales Sydney, Australia; ‡ St Vincent’s Centre for Applied Medical Research, Darlinghurst, Australia; § Cardiology Department, Royal Prince Alfred Hospital, Camperdown, Australia; ¶ Department of Cardiac and Thoracic Aortic Surgery, Medical University of Vienna, Austria; ∥ Victor Chang Cardiac Research Institute, Sydney, NSW, Australia; # Center for Medical Physics and Biomedical Engineering, Medical University of Vienna, Vienna, Austria; ** Ludwig Boltzmann Institute for Cardiovascular Research, Vienna, Austria.

**Keywords:** MCS, HM3, echo, hemodynamic, mock loop

## Abstract

The instantaneous left ventricular assist device (LVAD) flow waveform of the HeartWare ventricular assist device (HVAD) device was previously used to assess hemodynamic parameters, which is not possible with the HeartMate 3 (HM3). The purpose of this study was to assess the ability of pulsed wave Doppler interrogation of the outflow graft (PWOG) and of a novel noninvasive pump data acquisition system (HM3 Snoopy) to obtain noninvasive flow waveforms (NIFW) and to determine whether these predict hemodynamic changes. The study was conducted using a fluid-filled, biventricular failure mock loop model with a flow probe (FP) placed around the distal outflow. Five different pump speeds, two distinct hematocrits, and preload and afterload changes were used to create 56 unique hemodynamic states. Noninvasive flow waveform parameters were assessed for correlation against FP-derived parameters. Subsequently, NIFW parameters were assessed for their predictive capabilities for preload and afterload changes. There was moderate correlation between NIFW and FP-derived systolic waveform parameters (*r* range: 0.43–0.81), and strong correlation for diastolic parameters (*r* range: 0.87–0.99). Flow waveform amplitude as obtained by echocardiography was the best preload predictor (*r*^2^ = 0.67). A multiple linear regression model of NIFW parameters provided adequate prediction of afterload (*r*^2^ = 0.85). HeartMate 3 Snoopy and PWOG are promising tools for generating flow waveform surrogates and detecting hemodynamic changes.

## Graphical abstract

**Figure:**
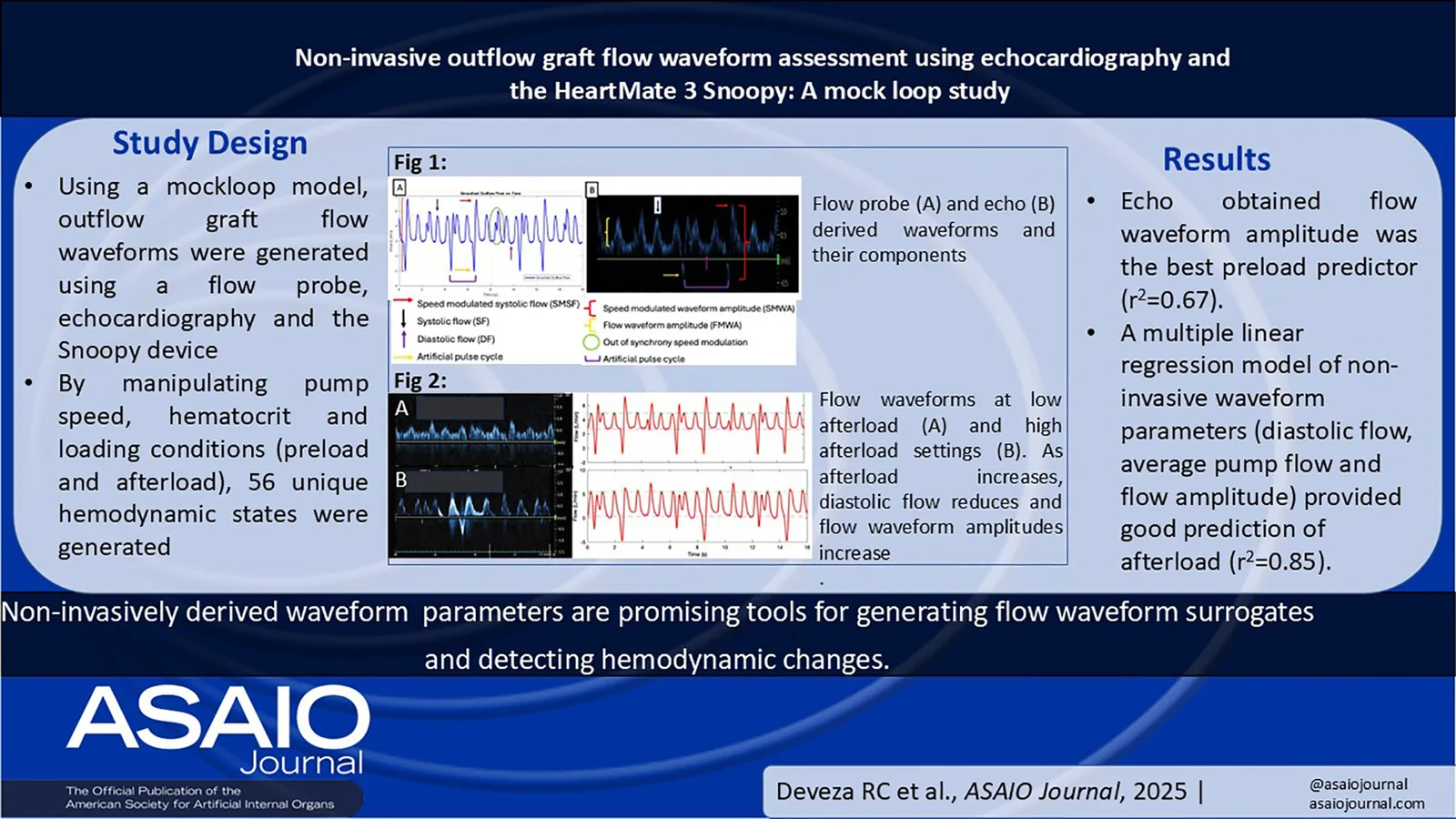

Left ventricular assist devices (LVADs) have become a well-established treatment modality for end-stage heart failure, both as a bridge to transplant and as destination therapy.^1^ Hemodynamic interactions between LVAD recipients and their device are dynamic and dependent on the loading conditions (*ie*, preload, afterload), pump speed settings, and intrinsic left ventricular function.^2,3^

Optimizing this complex patient-pump interaction remains an ongoing challenge for clinicians. General guidelines for LVAD recipient optimization include the use of echocardiography and right heart catheterization (with or without ramp tests^4^). The information obtained is used to guide speed settings and inform the use of diuretics and other heart failure therapies. Hemodynamic optimization of LVAD recipients has been shown to reduce hospitalization.^5,6^

The now-discontinued HeartWare HVAD (Medtronic, Mounds View, MN) provided an estimation of the instantaneous flow curve, which allowed clinicians to extract additional hemodynamic information based on waveform characteristics.^7–11^ High afterload states are associated with an increase in left ventricular (LV)-aortic gradient, which is most notable in diastole. This generates higher flow waveform amplitudes (FWAs), due to a reduction in diastolic flows (DFs), with potentially lower average flows. On the other hand, low afterload conditions are associated with a flatter, lower amplitude flow waveform secondary to a reduction in LV-aortic gradients affecting DFs preferentially, with a possible increase in average flow. Similarly, preload changes will affect LV-aortic gradients and average flow. High preload states correlate with higher flows and possibly increased waveform amplitudes, with low preload states having the opposite effect on the waveform characteristics.^7,12^ Additionally, the slope of the ventricular filling phase captured on the device monitor has been shown to correlate with pulmonary capillary wedge pressure (PCWP)^13^

The HeartMate 3 (HM3; Abbott Cardiovascular, Irvine, CA) is currently the only commercially available durable LVAD, supported by excellent outcomes relative to previous generation pumps.^14,15^ Unlike the HVAD, however, it does not provide real-time flow waveform monitoring. Pulsed wave doppler interrogation of the outflow graft (PWOG) of continuous flow devices has been used to assess various parameters of the LVAD flow waveform characteristics for the diagnosis of different conditions, such as aortic regurgitation and outflow graft malfunction.^16,17^ Parameters such as the systolic to diastolic ratio, systolic/diastolic slopes, and velocity–time integral have been demonstrated to correlate with pulmonary capillary wedge pressure.^16,18^

The available data on outflow graft waveform parameters for hemodynamic assessment, however, were obtained in older generations of pumps such as the HVAD and HeartMate II. To our knowledge, hemodynamic correlations between outflow graft waveform parameters and patient hemodynamics in HM3 recipients have not been systematically studied.

More recently, the HM3 Snoopy device, a pick-up coil that extracts additional data from the HM3 device, including estimated maximum and minimum pump flow, has shown promising results as an ancillary monitoring tool.^19^

The aim of this mock circulatory loop (MCL) study was two-fold. First, we sought to assess the correlations between flow probe-measured outflow graft waveform components and their corresponding noninvasively obtained components, using both PWOG and HM3 Snoopy. Second, we assessed the utility of these noninvasively obtained waveform parameters in detecting hemodynamic changes created in MCL, across a variety of loading conditions, hematocrits, and pump speeds.

## Methods

### Mock Circulatory Loop

**Figure 1.**
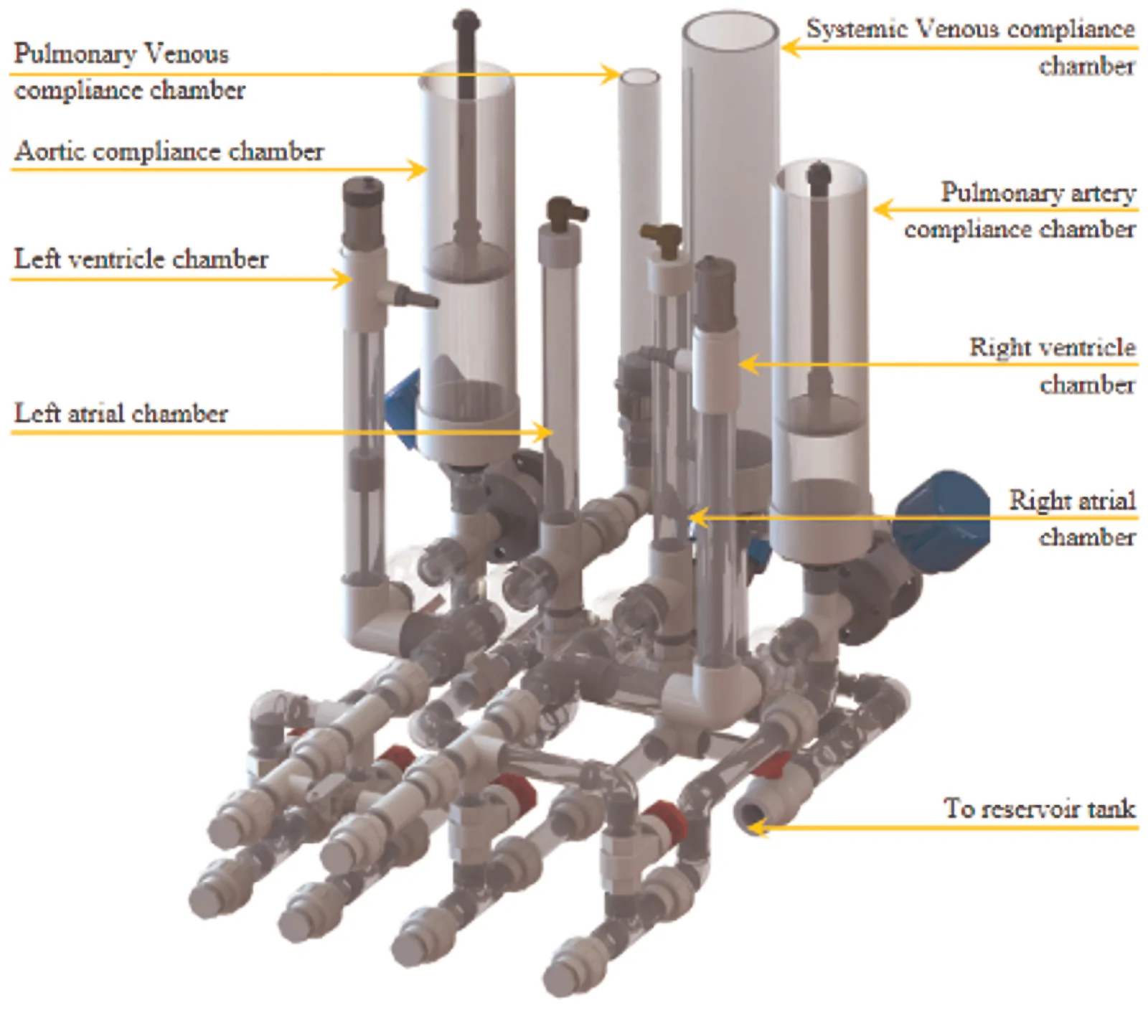
3D render of the MCL circuit. 3D, three-dimensional; MCL, mock circulatory loop.

The design of our MCL (Figure 1) has previously been described in detail.^20–22^ The MCL consists of four polyvinyl chloride (PVC) cylinders representing the right and left atria and ventricles. Mechanical check valves simulate mitral, aortic, tricuspid, and pulmonary valves to ensure unidirectional flow throughout the circuit. Proportional control valves manipulate systemic, pulmonary, and coronary resistance. Windkessel chambers simulate lumped systemic and pulmonary arterial and venous compliance. A water/glycerol 60/40% w/w solution was used as a blood analogue, at two different temperatures, 23°C and 29°C, approximating a hematocrit of 50% (viscosity of 3.3 mPa·s) and 40%(viscosity of ~2.8 mPa·s),^23^ respectively. The MCL was controlled by a model developed on a MATLAB/Simulink platform (MATLAB 2018b, The MathWorks Inc., MA), which was then deployed for real-time control using a dSPACE MicroLabBox (dSPACE GmbH, Paderborn, Germany). All data were acquired and recorded at 100 Hz through the MicroLabBox, generating 15 second files. All pressures were measured by Edwards pressure sensors (TruWave Edwards Lifesciences, Irvine, CA). Flows were measured by clamp-on ultrasonic sensors (central aortic, pulmonary arterial, and coronary vessels using Em-tec BioProTT).

**Figure 2.**
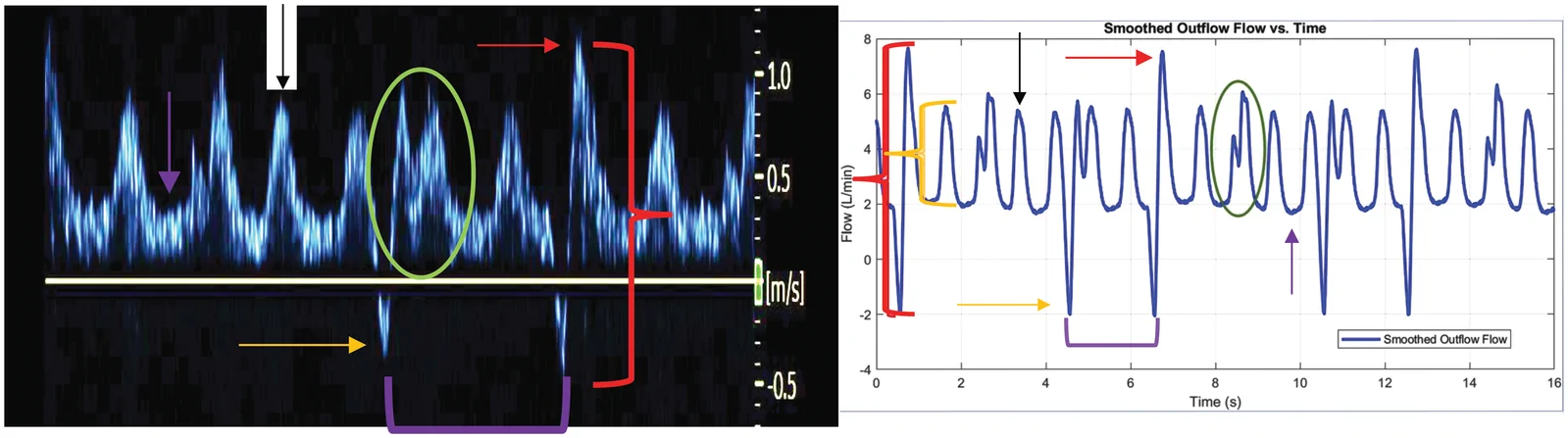
HM3 waveform components. (A), Flow probe-derived flow waveform. (B), Echo-derived velocity waveform.

The HM3 was connected to the distal region of LV PVC. An outflow graft fashioned with Tygon tubing (Saint-Gobain Performance Plastics, Germany), downsized to 10 mm diameter was connected to the aortic system. Outflow graft flow waveforms were assessed using a Transonics Clamp-on Flowsensor (FP). The following outflow graft flow waveform components were assessed: speed-modulated systolic flow (SMSF), systolic flow (SF), DF, speed-modulated diastolic flow (SMDF). From these basic parameters, additional components were generated: FWA (obtained by subtracting DF from SF), speed-modulated amplitude (SMA; obtained by subtracting SMDF from SMSF). These flow waveform components are similar to the ones obtained from the HVAD device, except for SMSF, SMDF, and SMA. When the HM3 device speed deceleration (caused by the intrinsic artificial pulse) aligns with ventricular diastole, it results in a pronounced dip in diastolic flow (SMDF). This event is then shortly followed by an increase in pump speed that occurs in synchrony with the beginning of systole, generating a spike in systolic flow (SMSF) (Figure 2, A and B). Left ventricular end diastolic pressure (LVEDP) was used as a surrogate for preload, whereas systemic vascular resistance (SVR) and mean arterial pressure (MAP) were the surrogates used for afterload.

### HeartMate 3 Snoopy

**Figure 3.**
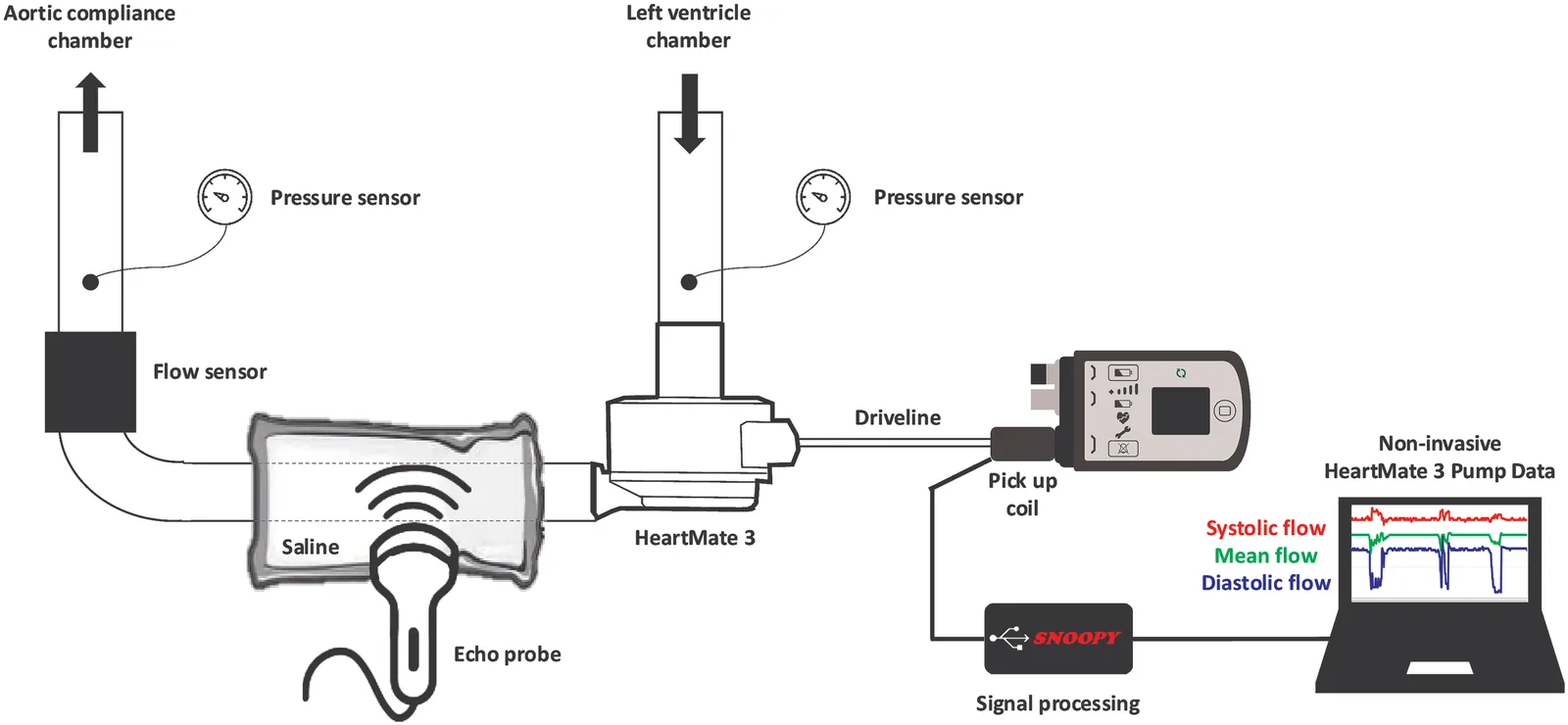
HM3 Snoopy components, user interface, and echocardiography setup. HM3, HeartMate 3.

The design and rationale for the HM3 Snoopy device have been published previously.^19^ In short, HM3 Snoopy is a magnetic pick-up coil mounted around the driveline (Figure 3). The communication between the pump and controller is based on request and response data. The induced voltage received is then converted to readable pump data, and the HM3 parameters are stored at 1 Hz (every second) and displayed via a graphical user interface (MATLAB, R2021b, The MathWorks Inc.). The twelve pump parameters collected by HM3 Snoopy are summarized in Table 1. Each hemodynamic setting generated by the mock loop was recorded for at least 30 seconds. The waveform parameters recorded were similar to the ones generated by the FP. It is important to note that HM3 Snoopy only captures single diastolic and systolic waveform components, which likely represent an averaged value of SF/SMSF and DF/SMDF. For the correlation analyses, HM3 Snoopy SF and DF were used as a surrogate against SMSF and SMDF comparisons, respectively.

**Table 1.** HM3 Snoopy Pump Main Monitoring Parameters (Per Second)

Parameters	Meaning
Speed (rpm)	Average pump rotor speed
Q mean (L/min)	Average estimated HM3 pump flow
Q min (L/min)	Minimum estimated HM3 pump flow
Q max (L/min)	Maximum estimated HM3 pump flow
Q pulse (FWA)	Estimated HM3 pump flow pulsatility (Qmax–Qmin)
PI	Pulsatility index

FWA, flow waveform amplitude; HM3, HeartMate 3.

### Pulsed Wave—Outflow Graft

Echocardiography (GE Vivid IQ, GE Medical Systems Information Technologies GmbH, Freiburg im Breisgau, Germany) with PWOG was used to generate velocity waveforms, with the sample volume placed inside the outflow graft. Two 500 ml saline bags surrounding the outflow graft were used to improve sound conduction (Figure 3). Two different insonation angles were used for each hemodynamic recording: a suboptimal angle (SA) and an optimal angle (OA). The SA was aimed to only allow for slight angulation from 90° relative to the outflow graft, as it approximates a common angle used for *in vivo* assessments of the outflow graft. The OA was obtained by optimizing the insonation angle as much as possible, given the spatial constraints.

All the waveform parameters obtained with the FP were also obtained with PWOG (Figure 2B). More specifically, PWOG SF and DF were generated by averaging three consecutive non-speed-modulated systolic and diastolic velocities. The highest SMSF and the lowest SMDF were recorded for each setting. The waveform amplitudes were obtained in the same way as described previously for the FP. Left ventricular assist device flow was calculated by averaging two 2 second velocity time integral (VTI) envelopes, then subsequently multiplying it by the cross-sectional area, then multiplying by 30 to convert flow into liters per minute.

### Study Protocol

The baseline condition for all experiments was a biventricular systolic heart failure model operating at 70 cycles per minute. Unique hemodynamic conditions were generated by systematically varying loading conditions (preload—baseline, high and low; afterload—baseline, high and low), pump speed (4,000, 4,500, 5,000, 5,300, 5,700 rpm), and hematocrit (40% and 50%). Loading conditions were modified electronically via proportional control valves that adjusted systemic and pulmonary resistance (to increase or reduce preload). Experiments began with a hematocrit of 50%, with loading conditions randomized and manipulated one variable at a time: preload was adjusted while maintaining baseline afterload, and vice versa. Once all possible preload and afterload permutations were completed, the pump speed was changed and the loading conditions were again altered, until all possible combinations were completed. This process was repeated for each of the five different pump speeds. The study protocol was then repeated at a hematocrit of 40%. For each hemodynamic condition, data were simultaneously recorded from the MCL, PWOG, and HM3 Snoopy.

### Statistical Analysis

Statistical analyses were performed using RStudio version 4.4 (Posit PBC, Boston, MA). Descriptive statistics were summarized as mean ± standard deviations. The hypothesis of normal distribution was tested using the Shapiro–Wilk test. Pearson’s correlation coefficient (*r*), including the 95% confidence interval, was calculated to evaluate dependencies between the flow probe-generated waveform component and the noninvasive-generated surrogates (PWOG and HM3 Snoopy). Spearman’s correlation coefficient (*r*) was used for non-normally distributed variables. Linear regression and multiple linear regression (*r*^2^) models were used to assess hemodynamic parameter changes. The variance inflation factor (VIF) was maintained below five for multiple linear regression models to limit collinearity.

## Results

A total of 60 unique hemodynamic scenarios were generated. Four of these scenarios were excluded as they generated nonphysiological (*ie*, negative) filling pressures, causing the mock loop simulation to collapse, with a remaining total of 56 scenarios available for analysis. Insonation angle for PWOG SA was (60.4° ± 6.6°), whereas PWOG OA was (42.7° ± 7°). A summary of MCL hemodynamic data is shown in Table 1, Supplemental Digital Content, https://links.lww.com/ASAIO/B710.

### Average LVAD flow correlations

There was a very strong correlation between the FP measured LVAD flow, PWOG SA (*r* = 0.9), PWOG OA (*r* = 0.86), and estimated mean HM3 flow assessed via HM3 Snoopy (*r* = 0.96).

### FP and noninvasive waveform components correlations

Moderate correlations between the systolic waveform parameters and the noninvasive modalities were found. With regard to diastolic parameters, on the other hand, there was excellent correlation. The performance of HM3 Snoopy and both PWOG angles was similar. Comparable levels of correlation were observed for FWA. Correlation levels for speed-modulated flow waveform amplitude (SMFWA) were affected by the angle of PWOG, with SA being worse than OA, which in turn was worse than HM3 Snoopy. Table 2 shows the three best noninvasive correlations for each waveform component.

**Table 2.** Waveform Components and Correlations of Noninvasive Modalities

Parameter	Modality	*r* Value	95% Confidence Interval	*p* Value
SF	PWOG OA	0.81	0.69–0.88	< 0.001
SF	PWOG SA	0.63	0.44–0.76	< 0.001
SF	HM3 Snoopy	0.55	0.33–0.72	< 0.001
SMSF	HM3 Snoopy*	0.69	0.52–0.8	< 0.001
SMSF	PWOG OA	0.52	0.30–0.68	< 0.001
SMSF	PWOG SA	0.43	0.19–0.63	< 0.001
DF	HM3 Snoopy	0.99	0.98–0.99	< 0.001
DF	PWOG OA	0.94	0.89–0.96	< 0.001
DF	PWOG SA	0.93	0.88–0.96	< 0.001
SMDF	HM3 Snoopy*	0.92	0.87–0.95	< 0.001
SMDF	PWOG OA	0.88	0.80–0.93	< 0.001
SMDF	PWOG SA	0.87	0.78–0.92	< 0.001
FWA	PWOG OA	0.94	0.90–0.97	< 0.001
FWA	HM3 Snoopy	0.91	0.85–0.95	< 0.001
FWA	HM3 Snoopy PI†	0.87	0.79–0.92	< 0.001
FWA	PWOG SA	0.8	0.68–0.88	< 0.001
SMFWA	HM3 Snoopy*	0.95	0.92–0.97	< 0.001
SMFWA	PWOG OA	0.77	0.64–0.86	< 0.001
SMFWA	PWOG SA	0.54	0.33–0.71	< 0.001

* HM3 Snoopy SF and DF were used against FP-generated SMDF and SMSF. Similarly, FWA was used as the comparator against SMFWA.

† PI was used as a surrogate for FWA.

DF, diastolic flow; FWA, flow waveform amplitude; LVEDP, left ventricular end diastolic pressure; MAP, mean arterial pressure; OA, optimal angle; PI, pulsatility index; PWOG, pulsed wave doppler of the outflow graft; SA, Suboptimal angle; SMDF, speed-modulated diastolic flow; SMFWA, speed-modulated flow waveform amplitude; SVR, systemic vascular resistance.

### Waveform Components and Hemodynamic Assessment

#### Preload (LVEDP)

There was a negative correlation between DF components and a positive correlation with waveform amplitudes. Table 3 shows the main results. In other words, the higher the preload, the larger the FWA, mainly due to a disproportionate reduction in the DF components. There were only weak correlations with systolic waveform parameters (Table 2, Supplemental Digital Content, https://links.lww.com/ASAIO/B711). Multiple linear regression analyses did not yield superior results to the individual waveform parameters.

**Table 3.** Waveform Components and Hemodynamic Correlations of Noninvasive Modalities

Parameter	Noninvasive Surrogate	*r* Value	95% Confidence Interval	*p* Value
	Negative Correlations			
LVEDP	PWOG OA SMDF	−0.69	−0.81 to −0.52	< 0.001
LVEDP	PWOG SA SMDF	−0.67	−0.80 to −0.50	< 0.001
LVEDP	HM3 Snoopy DF	−0.64	−0.78 to −0.45	< 0.001
LVEDP	PWOG OA DF	−0.62	−0.76 to −0.42	< 0.001
LVEDP	PWOG SA DF	−0.56	−0.72 to −0.34	< 0.001
SVR	PWOG OA SMDF	−0.69	−0.81 to −0.53	< 0.001
SVR	PWOG SA SMDF	−0.66	−0.78 to −0.48	< 0.001
SVR	PWOG OA DF	−0.54	−0.71 to −0.33	< 0.001
SVR	PWOG SA DF	−0.47	−0.65 to −0.22	< 0.001
SVR	HM3 Snoopy DF	−0.47	−0.66 to −0.23	< 0.001
MAP	PWOG OA SMDF	−0.59	−0.74 to −0.38	< 0.001
MAP	PWOG SA SMDF	−0.52	−0.69 to −0.30	< 0.001

	Positive Correlations			
LVEDP	PWOG OA FWA	0.81	0.70–0.89	< 0.001
LVEDP	PWOG SA FWA	0.78	0.65–0.86	< 0.001
LVEDP	HM3 Snoopy FWA	0.70	0.54–0.81	< 0.001
LVEDP	PWOG OA SMFWA	0.7	0.54–0.82	< 0.001
LVEDP	HM3 Snoopy PI	0.68	0.50–0.80	< 0.001
LVEDP	PWOG SA SMFWA	0.6	0.40–0.74	< 0.001
MAP	HM3 Snoopy SF	0.54	0.32–0.71	< 0.001
MAP	HM3 Snoopy FWA	0.69	0.52–0.81	< 0.001
MAP	HM3 Snoopy PI	0.48	0.24–0.66	< 0.001
MAP	PWOG OA SMFWA	0.60	0.40–0.74	< 0.001
SVR	HM3 Snoopy FWA	0.76	0.62–0.85	< 0.001
SVR	HM3 Snoopy PI	0.64	0.46–0.78	< 0.001
SVR	PWOG OA SMFWA	0.58	0.38–0.73	< 0.001
SVR	PWOG OA FWA	0.54	0.32–0.70	< 0.001

	Multiple Linear Regression	*r* ^2^		
MAP	HM3 Snoopy FWA, HM3 Snoopy average flow, and PWOG OA SMDF	0.85		< 0.001

* HM3 Snoopy SF and DF were used against FP-generated SMDF and SMSF. Similarly, FWA was used as the comparator against SMFWA.

DF, diastolic flow; FWA, flow waveform amplitude; LVEDP, left ventricular end diastolic pressure; MAP, mean arterial pressure; OA, optimal angle; PI, pulsatility index; PWOG, pulsed wave doppler of the outflow graft; SA, suboptimal angle; SMDF, speed-modulated diastolic flow; SMFWA, speed-modulated flow waveform amplitude; SVR, systemic vascular resistance.

#### Afterload (SVR and MAP)

Similarly, to what was observed with preload, as afterload increases, there is a reduction in diastolic waveform parameters with increases in afterload, as well as an augmentation of waveform amplitudes and pulsatility index (PI). These changes are illustrated in Figure 1, A–C, Supplemental Digital Content, https://links.lww.com/ASAIO/B709. Once again, systolic waveform parameters did not perform well, with the exception of HM3 Snoopy SF when MAP was used as the surrogate for afterload (Table 2, Supplemental Digital Content, https://links.lww.com/ASAIO/B711). Similarly to LVEDP, when SVR was used, multiple regression models using multiple waveform components did not generate superior results to single parameters. Table 3 summarizes the strongest correlations obtained. Conversely, a multiple linear model using HM3 Snoopy FWA, mean flow, and PWOG GA SMDF resulted in a significantly improved predictive ability for the MAP, with an *r*^2^ of 0.85, *p* < 0.001 (Table 3).

## Discussion

The use of flow waveforms as a hemodynamic tool was extremely useful but largely underused during the HVAD era. Although waveform parameters have been studied in HVAD and HeartMate II recipients in the context of hemodynamic assessment,^13,16,18,24,25^ these devices had more predictable flow waveforms. The HM3 device has a rather unique flow waveform due to its artificial pulse algorithm (Figure 2, A and B). Although some waveform parameters, such as SF, DF, and FWA, are present in all the older devices, the HM3 has unique waveform components, namely SMDF, SMSF, and SMFWA. To our knowledge, this is the first study to assess all those waveform parameters in a hemodynamic assessment context. In addition, this study expands on potential clinical uses for the HM3 Snoopy device.^19^

We systematically assessed the clinical utility of noninvasively assessed HM3 outflow graft flow waveforms and their hemodynamic correlations. Using pulsed flow waveform data, we were able to assess the interaction between pump flow and loading conditions for the first time in the HM3.

Our main findings are: 1) PWOG and HM3 Snoopy derived LVAD mean and minimum flows have strong correlation with FP measured LVAD flow; 2) PWOG and HM3 Snoopy diastolic measurements show very strong correlation against FP; 3) Correlation between noninvasive systolic parameters and hemodynamic changes is suboptimal (*r* values 0.43–0.81) 4) There is a strong correlation between noninvasive diastolic waveform parameters and hemodynamic changes (*r* values 0.87–0.92); 5) Increases in preload and afterload result in an increased FWA and a decrease in DFs; These waveform changes with different loading conditions is compatible which what was previously described empirically in HVAD recipients^7^; 6) In most scenarios, optimizing insonation angles improves the correlation of noninvasive parameters with FP recorded waveform parameters and hemodynamic changes.

We believe that the suboptimal correlation between the noninvasive modalities and the systolic waveform parameters can be partially explained by the relatively low variability of these parameters across various hemodynamic simulations (SF mean 5.01 ± 0.61 L/min; SMSF mean 7.66 ± 0.66 L/min) compared with the diastolic parameters (DF mean 1.83 ± 1.44 L/min; SMDF mean ‐2.62 ± 1.82 L/min). In addition, as the HM3 exhibits a curvilinear relationship between power and flow at higher flow rates, it consequently leads to an underestimation of peak SF values.^3^

Multiple linear regression models did not improve the predictive ability of isolated parameters for most hemodynamic variables. However, MAP variation was largely explained by a combination of HM3 Snoopy FWA, HM3 Snoopy average flow, and PWOG GA SMDF in a multiple linear regression, with an *r*^2^ of 0.85. This is particularly encouraging as blood pressure measurement in LVAD recipients remains a challenge due to low pulse pressure,^16^ and more accurate noninvasive tools would be a welcome addition. Further development of additional pump function assessment tools, such as Snoopy, may allow noninvasive assessment of blood pressure.

## Limitations

Our study has limitations that should be discussed. First, this is an *in silico* investigation, utilizing a biventricular model with isolated ventricles and glycerol as a blood substitute. This configuration does not allow for ventricular interdependence to be studied. Furthermore, the results and correlations obtained in this study cannot be immediately translated to clinical practice without a human study confirming these findings.

Second, whereas PWOG was readily obtained in this study due to direct visualization and manipulation of the outflow graft, identifying the outflow graft in clinical practice is often challenging. Additionally, we used a 10 mm diameter outflow graft, whereas the HeartMate outflow graft diameter is 14 mm, which affects hydraulic resistance and fluid inertia,^26^ as well as velocities inside the conduit.

Third, tracing PWOG envelopes is operator dependent, and this technique could be potentially affected by inter-operator variability. We also observed more noise in this *in silico* acquisition of PWOG compared to *in vivo* assessments, likely due to the presence of bubbles mixed with glycerol in the MCL circuit. The available human data are encouraging, with high success rates and low interobserver variability.^16,18^ It should be noted that these studies were performed in highly specialized centers.

Lastly, the human circulation and the MCL are a series circuit. In this scenario, it is impossible to modulate preload without affecting afterload, and vice versa (Table 1, Supplemental Digital Content, https://links.lww.com/ASAIO/B710). With that said, our study was able to identify the main waveform changes that occur when preload or afterload are preferentially affected.

## Conclusions

In this MCL study of the HM3 LVAD, noninvasively obtained DF waveform components showed significant correlations against FP-derived parameters. Additionally, these noninvasively assessed diastolic parameters have been shown to correlate well with hemodynamic changes, in keeping with previously published literature. We believe these new noninvasive tools could be clinically useful by helping to obtain a more refined assessment of LVAD recipients’ hemodynamics. We envision that in clinical practice, serial recordings of the outflow graft waveform parameters could inform therapeutic decisions, such as the use of diuretics, afterload reduction through intensification of heart failure therapy, and pump speed changes. *In vivo* studies testing these noninvasive modalities are warranted to further assess the clinical utility of these tools.

## Supplementary Material
