# Supplementary material for: Noninvasive Outflow Graft Flow Waveform Assessment Using Echocardiography and the HeartMate 3 Snoopy: A Mock Loop Study
Source: ASAIO J. 2025 Dec 22;72(10):812–8. doi: 10.1097/MAT.0000000000002610 (PMC13608275; doi:10.1097/MAT.0000000000002610)
Supplement: Supplementary file 1 [file mat-72-0812-s001.pdf]

Supplemental Table 2: Systolic waveform components and hemodynamic correlations of non-invasive modalities
SF: Diastolic flow
LVEDP: Left ventricular end diastolic pressure
MAP: Mean arterial pressure
OA: Optimal angle
PWOG: Pulsed wave doppler of the outflow graft
SA: Suboptimal angle
SMSF: Speed modulated systolic flow
SVR: Systemic vascular resistance

Parameter	Non-invasive surrogate	r value	95% Confidence Interval	p value
**LVEDP**	PWOG OA SMSF	0.24	-0.0022 to 0.48	NS
**LVEDP**	PWOG SA SMSF	0.06	-0.21 to 0.32	NS
**LVEDP**	HM3 Snoopy SF	-0.1	-0.35 to 0.17	NS
**LVEDP**	PWOG OA SF	0.4	-0.16 to 0.6	0.002
**LVEDP**	PWOG SA SF	0.23	-0.03 to 0.47	NS
**SVR**	PWOG OA SMSF	-0.01	-0.28 to 0.25	NS
**SVR**	PWOG SA SMSF	0.02	-0.24 to 0.29	NS
**SVR**	PWOG OA SF	0.08	-0.19 to 0.33	NS
**SVR**	PWOG SA SF	-0.06	-0.31 to 0.21	NS
**SVR**	HM3 Snoopy SF	0.26	-0.0023 to 0.29	NS
**MAP**	PWOG OA SMSF	0.2	-0.07 to 0.44	NS
**MAP**	PWOG SA SMSF	0.02	-0.24 to 0.28	NS
