# Supplementary material for: Noninvasive Outflow Graft Flow Waveform Assessment Using Echocardiography and the HeartMate 3 Snoopy: A Mock Loop Study
Source: ASAIO J. 2025 Dec 22;72(10):812–8. doi: 10.1097/MAT.0000000000002610 (PMC13608275; doi:10.1097/MAT.0000000000002610)
Supplement: Supplementary file 2 [file mat-72-0812-s002.pdf]

Preload setting	Afterload setting	LVEDP (mmHg)	SVR (dynes/sec)	MAP (mmHg)	FP measured LVAD flow (L/min)
Baseline	Baseline	16.0 ± 3.8	1552.5 ± 689.1	84.5 ± 14.4	3.1 ± 0.9
Baseline	Increased	23.6 ± 2.3	3380.6 ± 816.8	110.7 ± 15.6	1.8 ± 0.8
Baseline	Reduced	13.4 ± 2.5	1457.9 ± 351.8	78.7 ± 13.2	3.2 ± 0.4
Increased	Baseline	18.8 ± 4.2	977.9 ± 118.2	83.0 ± 5.5	3.5 ± 1.1
Reduced	Baseline	8.72 ± 3.26	794.5 ± 94.7	58.8 ± 12.3	3.1 ± 0.4

Supplemental Table 1: Main parameters recorded on the MCL across different hemodynamic settings. The values are reports as average ± standard deviation.
LVEDP - Left ventricular end diastolic pressure
SVR – Systemic vascular resistance
MAP – Mean arterial pressure
FP – Flow probe
