# Supplementary material for: Noninvasive Outflow Graft Flow Waveform Assessment Using Echocardiography and the HeartMate 3 Snoopy: A Mock Loop Study
Source: ASAIO J. 2025 Dec 22;72(10):812–8. doi: 10.1097/MAT.0000000000002610 (PMC13608275; doi:10.1097/MAT.0000000000002610)
Supplement: Supplementary file 3 [file mat-72-0812-s003.pdf]

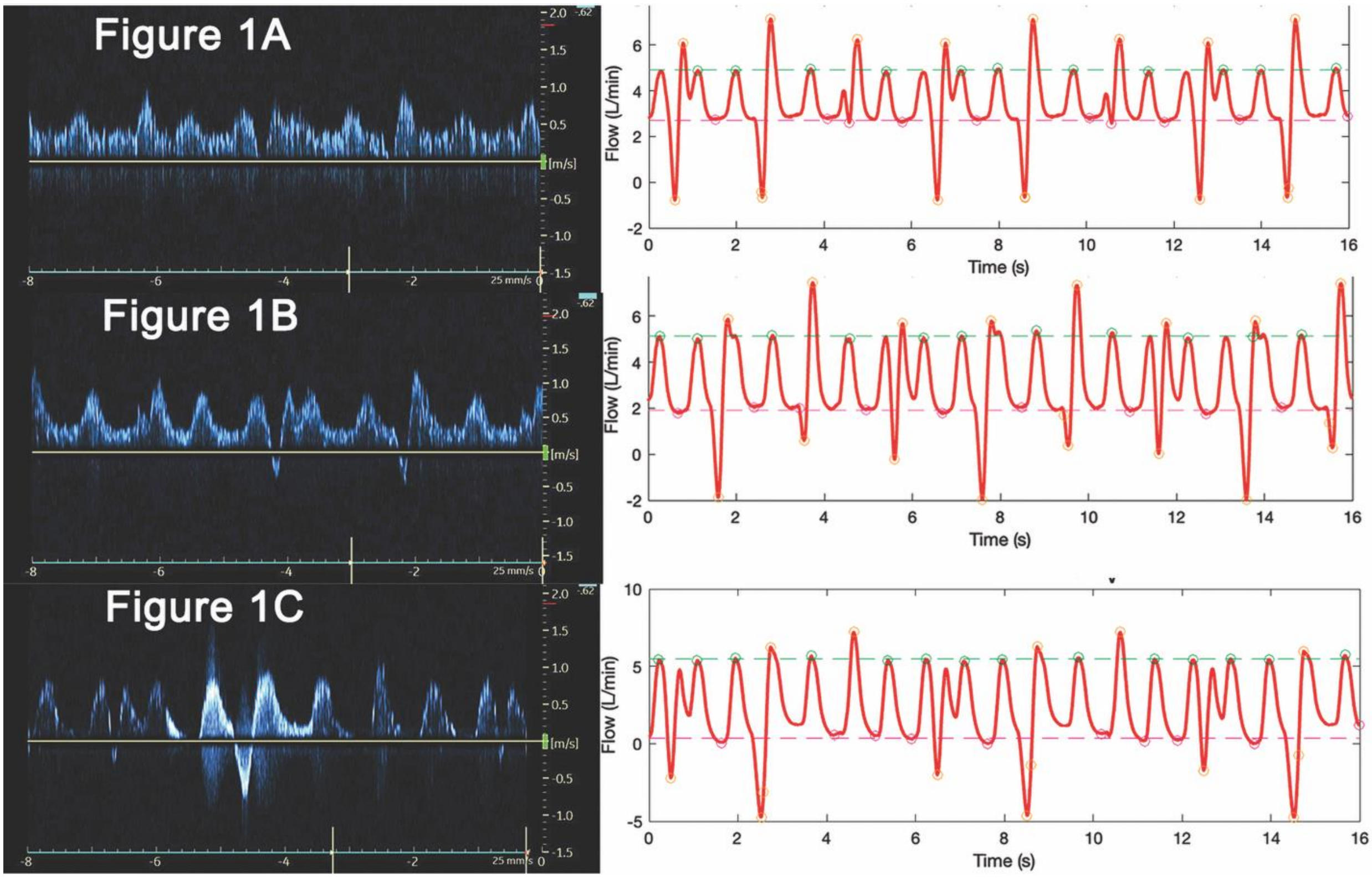

Supplemental figure 1: Pulsed wave doppler traces and their corresponding probe flow waveform recording with varying afterload settings. The traces highlight the inverse relationship between afterload and diastolic flow.

Figure 1A: Baseline preload, low afterload

- Average systolic flow: 4.91 L/min
- Average diastolic flow: 2.70 L/min
- Flow waveform amplitude: 2.21 L/min

Figure 1B: Baseline preload, baseline afterload

- Average systolic flow: 5.18 L/min
- Average diastolic flow: 1.90 L/min
- Flow waveform amplitude 3.28 L/min

Figure 1C: Baseline preload, high afterload

- Average systolic flow: 5.49 L/min
- Average diastolic flow: 0.37 L/min
- Flow waveform amplitude 5.12 L/min
